# Social interaction, languaging and the operational conditions for the emergence of observing

**DOI:** 10.3389/fpsyg.2014.00899

**Published:** 2014-08-14

**Authors:** Vincenzo Raimondi

**Affiliations:** Linguistique Anthropologique et Sociolinguistique – Institut Marcel Mauss, École des Hautes Études en Sciences SocialesParis, France

**Keywords:** social interaction, recursive consensual coordination, languaging, observing, bio-logical approach, Maturana, Tomasello, intention-reading

## Abstract

In order to adequately understand the foundations of human social interaction, we need to provide an explanation of our specific mode of living based on linguistic activity and the cultural practices with which it is interwoven. To this end, we need to make explicit the constitutive conditions for the emergence of the phenomena which relate to language and joint activity starting from their operational-relational matrix. The approach presented here challenges the inadequacy of mentalist models to explain the relation between language and interaction. Recent empirical studies concerning joint attention and language acquisition have led scholars such as Tomasello et al. (2005) to postulate the existence of a universal human “sociocognitive infrastructure” that drives joint social activities and is biologically inherited. This infrastructure would include the skill of precocious intention-reading, and is meant to explain human linguistic development and cultural learning. However, the cognitivist and functionalist assumptions on which this model relies have resulted in controversial hypotheses (i.e., intention-reading as the ontogenetic precursor of language) which take a contentious conception of mind and language for granted. By challenging this model, I will show that we should instead turn ourselves towards a constitutive explanation of language within a “bio-logical” understanding of interactivity. This is possible only by abandoning the cognitivist conception of organism and traditional views of language. An epistemological shift must therefore be proposed, based on embodied, enactive and distributed approaches, and on Maturana’s work in particular. The notions of *languaging* and *observing* that will be discussed in this article will allow for a bio-logically grounded, theoretically parsimonious alternative to mentalist and spectatorial approaches, and will guide us towards a wider understanding of our sociocultural mode of living.

## SOCIAL COGNITION AND LANGUAGE

Over the last decades, “social cognition” has become the object of intense interdisciplinary research. Many theoretical and empirical efforts have been dedicated to understanding the specific conditions on which human interaction and the ontogenetic development of our socio-interactional skills rely. In this context, explaining how individuals involved in interaction solve the “problem of other minds” in order to conduct effective coordination stands out as a major challenge for many scholars. However, a debate has flourished concerning the validity of supposing some kind of “mindreading” to account for social interaction. Whereas the cognitivist accounts view this as a crucial issue (e.g., Frith, 2008) and propose several models to resolve it, the embodied and enactive approaches consider representational and spectatorial explanations of human interactivity to be inadequate. According to the latter, social engagement with others does not fundamentally constitute a cognitive problem to be solved through the mutual detection of mental states by the interacting individuals; rather, it is the result of embodied, ecologically embedded, intersubjective dynamics (De Jaegher and Di Paolo, 2007; Gallagher, 2008a,b; Hutto, 2009; De Jaegher et al., 2010; Di Paolo and De Jaegher, 2012).

Consistent with non-mentalist approaches to interaction, I would like to direct our attention to how the explanation of linguistic activity can broaden our understanding of human interaction and sociality. Up to the present, theories in the cross-disciplinary domain of social cognition have not privileged the investigation of the linguistic phenomenon, or have taken traditional views of language for granted. A partial exception to this is Tomasello’s influential research conducted on joint activity, leading to the author’s hypothesis of a *functional* relation linking intention-reading to language, and language acquisition in particular. However, this hypothesis is questionable, as is Tomasello’s conception of language.

A major obstacle for understanding the *constitutive* relation that links language to social interaction is the fact that the linguistic phenomenon is still frequently conceived in inadequate terms. Here I will propose an alternative explanation of both language and social interaction using a different epistemological framework. To this end, I will first draw on Tomasello’s model to discuss the limits of cognitivist approaches, including those that are more “socioculturally oriented.” I will subsequently show how these limits can be overcome.

Building on developmental and comparative research, Tomasello et al. (2005) offer an interdisciplinary approach in order to explain language and culture by tracing them to the foundational conditions of social engagement and joint activity (e.g., Carpenter et al., 1998; Tomasello, 1999, 2003). According to Tomasello, both human collaborative activities and communication – conceived as a special activity based on the utilization of “linguistic symbols” as cultural artifacts – are possible thanks to our prosocial dispositions and certain unique cognitive skills. Modified throughout the years, the most recent version of this theory downplays the simulationist positions previously held by Tomasello (1999) and postulates that a species-specific *sociocognitive infrastructure* provides humans with the capacity for “shared intentionality”^1^ (Tomasello et al., 2005; Tomasello, 2008). Along these lines, Tomasello puts forth the theory of a universally inherited infrastructure which would include skills for imitative learning and role-reversal, a disposition for cooperation and the uniquely human skill of recursive intention-reading, allowing us to understand communicative intentions cooperatively. In language sciences, similar arguments have been proposed by Levinson, among others, in his hypothesis of an innate and universal “interaction engine” (Levinson, 2006a,b).

Supported by a host of experiments, Tomasello’s theory is supposed to account for, among other things, the ontogenetic emergence of “joint attention” in infants’ early interactions. Beginning around nine months of age, infants start to jointly attend to objects with others in interactive settings, following the other’s gaze (Scaife and Bruner, 1975; Bruner, 1977), and starting to respond to and initiate pointing gestures (Bates et al., 1975). While the explanation of the emergence of such “triadic” interactions is the object of fierce debate (see, e.g., Eilan et al., 2005; Seemann, 2011), Tomasello, in agreement with Bruner’s (1995) conception of just such a developmental step as the first “meeting of minds,” argues that the emergence of joint attention reveals the development of intention-reading skills, permitting the child to “know together” with his caregivers that they are attending to the same thing (Tomasello, 2008). This is supposedly the first step in the subsequent development of full-fledged mindreading (Lohmann et al., 2005; Tomasello et al., 2005).

What then is the impact of this hypothesis on our understanding of language? Tomasello argues that not only could the hypothesis of a sociocognitive infrastructure explain language acquisition, it could also offer important insights for comparative research as well as phylogenetic investigation into the origins of language. The crucial point here is that the conventionalized symbolic system which we use to coordinate with each other in joint activities, or “linguistic code” as it is labeled by Tomasello, “(…) rests on a nonlinguistic infrastructure of intentional understanding and common conceptual ground, which is in fact logically primary” (Tomasello, 2008: 58). By discovering the communicative intentions of the others, the child ontogenetically acquires skills for communication, typically by first understanding and initiating activities based on joint attention (for example, by pointing at objects in order to request them), and then by appropriating intention-based expressions addressed to him by adults. In this manner, precocious intention-reading gradually allows the child to grasp the meaning and function of conventional symbols, which can be then mapped into usage-patterns (Tomasello, 2003). In other words, Tomasello’s model supposes that shared understanding of goals and recursive intention-reading are already in place when children begin to speak. According to this model, the sociocognitive infrastructure is a prerequisite for language acquisition and is, in fact, its developmental precursor. In line with this, Tomasello recommends that studies on the phylogenetic origins of both language and cultural life should include an inquiry into the evolution of this sociocognitive infrastructure as a necessary preadaptation for the emergence of language and culture. Moreover, he argues that qualitative differences between contemporary primates with regard to social engagement and symbolic communication would be explained by the hypothesis that non-human primates lack just such a species-specific skill enabling the detection of communicative intentions in a cooperative goal.

It is beyond the scope of this paper to offer an exhaustive analysis of Tomasello’s theory, so I will not be able to address all of its important insights concerning cooperation and human sociality (e.g., Tomasello, 2009, 2011). I will restrict myself to discussing the explanation provided for interaction and language through the notion of intention-reading, in order to present a non-mentalist approach to the same questions.

*Prima facie*, looking to social interaction and joint activity in order to seek out the *raison d’être* of language may not seem problematic in itself; quite the contrary. As opposed to formalist and nativist views of language, the conception of linguistic phenomena as inherently social and activity-grounded can be linked to several long-standing positions held both in linguistics and philosophy. Undoubtedly, any theorization about the precise conditions necessary for language to emerge within interactional real-time dynamics – which is admittedly one of the principal aims of Tomasello’s work – is a precious contribution.

However, when it comes to the hypothesis provided, Tomasello’s model remains highly contentious. First of all, Tomasello’s position has garnered criticism concerning the postulated precocious emergence of intention-reading, as well as the complex meta-representations and recursivity it would entail (Griffin and Dennett, 2008; Moore and Barresi, 2010; Reboul, 2010). Another controversial issue concerns the idea that a communicative intention could be understood independently from the precise linguistic forms that express it; by definition, one cannot come without the other (for a similar argument, see Taylor and Shanker, 2003). Tomasello actually argues in favor of a causal relation between a communicative intention and its linguistic form, in that the grasping of the former leads to the subsequent appropriation of the latter. However, although Tomasello claims to draw on philosophy of language for such notions as “non-natural meaning” (Grice, 1989) and communicative intention, it should be observed that the theories to which he refers do not imply the “developmental claim that an understanding of intentions comes before communication” (Racine, 2011: 33). In addition to this, and more importantly, Tomasello offers no operational explanation for the emergence of any mechanism of intention-reading; it is merely assumed to exist, as though it were a “X-ray perception” of intentions (Cowley, 2004). For this reason, I contend that this mechanism is not at all operationally grounded. The emergence of such a functional skill remains *unexplained*, although seemingly *justified* by its putative function in bio-logical heritage as sort of cognitive leap separating humans from other primates (Raimondi, 2013). Based on our knowledge of living beings, what operational foundation would allow the assumption that a human organism could develop such a mechanism by the age of nine months? One of the main limits of the hypothesis is that an intention-reading mechanism should be explained starting from its own conditions of possibility. However, as soon as we try to show its emergence, we become aware that precocious intention-reading is neither operationally possible nor necessary.

While Tomasello rejects the existence of a Chomskian linguistic faculty, he proposes a sociocognitive infrastructure based on a similar conception of organism and ontogenetic development. Ultimately, Tomasello’s model relies on highly questionable assumptions about the status of language as a symbolic conventional tool and the role of mind in the explanation of interaction. The hypothesis of intention-reading as a precursor to linguistic learning is therefore dependent on controversial epistemological background.

I would therefore suggest a shift in focus to address the issue of the constitutive relation between interaction, joint activity and language on radically different epistemological bases. On the one hand, I will challenge Tomasello’s conception of mind, interaction and language. On the other hand, I will propose alternative theoretical arguments to show that language and human interaction are not functionally but constitutively related as they take place in the same operational-relational matrix. This means that we need to show how individuals, through the operation of mutual coupling, generate the interindividual domain to which linguistic and interactional phenomena should be traced in order for them to be explained. By the same token, it will become possible to understand why we cannot consider such phenomena to be the product of any faculty or property of the mind, precluding any mentalist explanation to account for their generation.

## INTERACTION, SEE UNDER MIND

Along with others scholars (De Jaegher and Di Paolo, 2007; Gallagher, 2008a,b; Leudar and Costall, 2009; De Jaegher et al., 2010; Di Paolo and De Jaegher, 2012), I argue that cognitivist approaches are inadequate to provide an explanation of social interaction. I discuss some of the issues related to such approaches by drawing on Tomasello’s model. After all, the sociocultural approach which Tomasello seeks to provide does not prevent him from relying on a conception of “mind” that, however “socially oriented,” remains committed to the some traditional cognitivist assumptions about mind and behavior. Epistemogically, this model endorses mentalist and folk-psychological views of organism as well as a spectatorial conception of interaction.

Mentalist assumptions include the idea that all phenomena related to the individual’s interactions with his environment could be explained by the presence of a mental mechanism which would be functionally responsible for the generation of said phenomena (in the present case, Tomasello’s recursive intention-reading is such a mechanism). This supposes a hierarchical organization inherent to the organism whereby phenomena belonging to the behavioral level arise*as specified* by processes taking place at another level, whether the latter be mental mechanisms or the neurobiological implementation of these mechanisms. Cognitive mechanisms are therefore assumed to be endowed with causal powers in the generation of behavior. Accordingly, they determine the adaptive competence of the organism that interacts with its medium. Such a hierarchical relation between mind and behavior is thus viewed as fundamental. This is consistent with the representationalist conception of cognition as an internal process that generates a representation of the environment in order produce an adequate response to it. Within this tradition of thinking, since subpersonal operations supposedly explain the organism’s “know-how,” mentalist explanations seem to be a suitable way to account for interactional phenomena.

By folk-psychological characterizations of mind, I refer to the pervasive idea that intentions and other mental states, normally ascribed to agents in daily life, are entities that exist on a more fundamental level than the behaving agents themselves. For example, Tomasello et al. (2005) endorse a mentalist and folk-psychological view of cognition in assuming that intentions and goals drive the genesis of behavior that is adaptive to the sociocultural niche. From this perspective, “intention” is actually conceived as an “internal entity that guides the person’s behavior” (Tomasello et al., 2005: 676).

Mentalist and folk-psychological views of cognition are intimately connected to an intellectualist postulate which assigns a spectatorial position to interacting individuals. According to such a view, these interacting individuals are being constantly faced with the problem of mutually detecting and predicting the mental states underlying the other’s behavior. Because of this assumption, Tomasello argues that shared intentionality, as the foundation of joint activity and communication, can only be achieved through special skills allowing the comprehension of others’ cooperative intentions. The spectatorial view implies that the agent needs to represent the others’ minds in order to achieve intersubjectivity with them. Since intentions are supposedly internal entities that cause behavior, a child is immediately faced with the problem of making sense of the behavior of adults. Before he can grasp intentions, “(…) from the infant’s point of view the adult is just making noise (for whatever reason)” (Tomasello, 2003: 23). Therefore, bridging the *self/other gap* requires an *ad hoc* infrastructure. However, this functionalist explanation relies on the creation of a mechanism coherent with the problem that the analyst himself posits as such.

By drawing on Tomasello’s model, I have briefly illustrated some of the epistemological reasons why many studies of social cognition consider human beings to be spectators of others’ behavior, and focus on individual mechanisms in order to explain how we act together and understand each other in interactive settings. However, I contend that these assumptions are based on an inadequate conception of organism, and that cognitivist heuristics unavoidably lead to a one-dimensional, individually-grounded notion of interaction. It should be remarked that the conflation of interactional and individual in the cognitivist approach causes us to lose sight of the interactional as a distinct domain.

## THE EPISTEMOLOGICAL BACKGROUND FOR A BIO-LOGICAL EXPLANATION OF INTERACTION

As an alternative epistemological paradigm, I will rely on Maturana’s “Biology of cognition” (Maturana, 1978, 1988, 2002), and on some assumptions shared by embodied and enactive approaches. In the interest of brevity I will only highlight certain aspects of Maturana’s theoretical contribution and I will assume that most of its core features (e.g., autopoietic organization, structural determinism, nervous system’s operational closure etc.) are already familiar to the reader, as well as its similarities and differences with regards to the enactive and embodied approaches.

What I define hereafter as a “bio-logical approach” is based on just such a non-reductionist epistemological framework. In a nutshell, taking a bio-logical stance to account for interaction means seeking out the *conditions of possibility* for all phenomena related to interacting individuals by drawing on our understanding of living beings. To this end, we need to make explicit the systemic conditions under which social interaction exists, clarify its relation with the constitution of living beings, and provide it with a generative explanation. By “generative explanation,” I mean an explanation that first traces the phenomena requiring explanation to the existential domain where they belong, and then proposes a mechanism that generates the *explanandum*. In this case, the phenomena to be explained are social interaction and language.

The bio-logical approach challenges the traditional cognitivist view of living being. Whereas the latter takes for granted a hierarchical organization (wherein the neurobiological level determines and controls the behavioral level, as we have seen above), the former posits two non-hierarchically related domains: on one hand, the domain of the living being’s structural components, and on the other, the domain in which the living being exists as an organism. Like every system, living beings basically exist as such in two co-occurrent domains: one in which it can be seen as an organism operating *as a whole* in interaction with its medium; and one in which it exists as a *composite entity* which can be deconstructed in order to observe its molecular and supramolecular components, its internal dynamics, and its structural changes. As Maturana argues, these two domains “do not intersect”: they constitute two radically different domains of phenomena that cannot be reduced to each other. Consequently, any attempt to explain the phenomena of one domain in terms of the other is inadequate. There is, however, a dynamic generative relation between them arising from the structural changes that the living being and its medium trigger in each other during the course of their “structural coupling” (see, e.g., Maturana et al., 1995).

Let us examine what adopting this view implies. On one hand, neurobiological processes belong to the domain of structural components. On the other hand, the apparent and non-apparent *dimensions* of the *relational operation* of the living being with its medium, such as behavior, mind, and emotions, constitute the “operational sphere” of the organism as a whole, and cannot be traced to the domain of components. Although the structural dynamics that takes place in the domain of the components participate in the systemic process, these dimensions pertain to the organism as a whole and denote classes of phenomena that take place in the operational domain in which the individual exists as such. Strictly speaking, such dimensions are determined neither by the system’s structure (the “inside”) nor by the medium’s structure (the “outside”), but are dependent on the dynamic interplay between the two. However, this co-modulation is constrained by the structures of both the organism and the medium. The result of this structurally determined dynamic is the generation of the operational relational matrix in which the organism exists at every moment in the course of its living as a spontaneous outcome of both a phylogenetic and ontogenetic history. The *organism’s existential domain* is therefore *inherently operational and relational*.

Several conclusions can be drawn from this approach. First, it prevents us from assuming a neurocentric conception of cognition. Cognition concerns the organism as a whole, not its components. Maturana and Varela (1980, 1992) have shown that the neural network operates as a closed system and does not have inputs and outputs, properly speaking. For that reason, the nervous system does not and cannot pick up information from the environment in order to compute a representation of it, nor can it specify the phenomena taking place in the domain of the organism as a whole. The role and the adaptive character of neurobiological processes in the generation of the organism-as-a-whole’s relational operation are to be understood as part of a systemic, dynamic process that involves both the operations of the organism and the medium (see, e.g., Maturana, 2000). This dynamic triggers structural changes in both the living being and its medium in such a manner that they cannot be anything but congruent to each other until the living being dies.

Second, this approach prevents us from accepting mentalist explanations. Unlike the traditional cognitivist position, the bio-logical framework allows the relation between different dimensions of the individual’s operational sphere, such as those of behavior and mind, to be understood in terms of systemic solidarity; that is to say, one dimension does not specify the features of another, neither do the different dimensions “exert a control” over each other. In other words, within the organism’s operational sphere, no dimension is to be considered as more fundamental than the others. However, the multidimensional architecture of the organism’s operational sphere and its constitutive systemic dynamics allows us, as observers, to establish correlations between its different dimensions. As a matter of fact, if behavior, mind and emotion are different yet interdependent dimensions of the organism’s operational sphere, they could be conceived as Borromean rings, simultaneously distinct and interlocking.

Finally, since the mind is a dimension of the operation of the organism as a whole (and therefore does not coincide with neurobiological processes), and since the nervous system cannot be said to determine the generation of the organism’s operation, no linear causal power concerning the generation of behavior can properly be assigned to brain or mind, as is the case in mentalist approaches. Furthermore, intentions and goals belong to our description of the organism’s operational sphere in relation to its medium, and not to neurobiological processes. At the same time, it is clear that rejecting the Cartesian conception of mind does not imply that one subscribes to any kind of eliminativism or physicalism. Rather, it suggests that, as Keijzer (2001: 33) argues, “mind applies at a personal level and does not provide a conceptual framework which specifies how subpersonal processes operate to bring a person’s behavioral capacities into being.” To this we can add that operational-relational capacities are brought into being not by neurobiological processes alone, but by the dynamic interplay between these processes and the medium.

By understanding that the organism’s existential domain should be regarded as inherently operational and relational, it becomes possible to see all phenomena related to an organism’s relational operation as belonging to the domain of its realization as a whole. Social interaction, joint activities and language are not explainable as products of neurobiological dynamics or other inner mechanisms, since they take place in the relational domain. Thus, their emergence and specific features can only legitimately be explained with reference to the human operational–relational matrix.

## THE DOMAIN OF INTERACTION AND COORDINATION

Based on the bio-logics of living beings, what are the conditions through which human social interaction emerges and how are these conditions linked to language? Concerning interaction, I would like to emphasize that the bio-logical approach allows us to shift from an explanation of interaction centered on individuals to an explanation of interaction within its own domain as such. In focusing on the relational domain of interaction, we are aware that although this domain is brought forth through the operation of two or more organisms conserving their independent identities, it possesses its own organization. This approach radically challenges the individualist understanding of interactivity, and puts the interactional process at the heart of the present inquiry.

Let us begin by developing an explanation of interaction that will draw on the bio-logical standpoint. As seen before, the organism as a whole is structurally coupled to its medium, and the mutually adaptive relation between the two is an existential condition that results from a specific ontogenetic and phylogenetic history. Most importantly, the organism as a whole exists precisely through the *relational operation* of coupling. The relational operation is thus not episodic – rather it is brought forth by an ongoing, necessarily continuous dynamic. Interaction between organisms can therefore be better understood as a spontaneous and inevitable consequence of structural coupling; that is to say, as a recurrent event in the ontogenetic history of living beings^2^. It follows that our understanding of interaction is logically subordinated to our understanding of the constitutive conditions of structural coupling. In other words, in accordance with Maturana and Varela, we can say that interaction is subordinated the conservation of the invariant conditions of living: that is to say, the *autopoietic organization* of living being (which takes place in the domain of components) and the organism’s *relation of adaptation* to its medium (which takes place in the domain of the organism as a whole). In other words, we do not need to provide any justification for the fact that interactions happen all the time throughout the biosphere, nor for the effectiveness of these interactions. What is needed is instead to identify the conditions that generate different interactional phenomena among different species in general, and joint activity amongst human beings in particular.

It is clear that from a non-representationalist point of view, interaction can often be analyzed as a bi-directional, co-regulated dynamic of coordination, as shown by theorists of both dynamics systems and enactive approaches (e.g., Fogel, 1993a,b; De Jaegher and Di Paolo, 2007; Fogel and Garvey, 2007). In line with Maturana’s definition, I argue that we can speak of *consensual coordination* when:

(1) during an event of interaction, we can distinguish an unfolding sequence of interrelated operations which are evidence of an interdependence between the operational spheres of individuals involved;
(2) these patterns of interrelated operations are the spontaneous result of a specific history of interaction and are inherently contingent on that peculiar co-ontogenetic history;
(3) the consequences of such an event on the respective operational spheres result in subsequent interactions.

Thus defined, consensual coordination is similar to the ethological notion of “ontogenetic ritualization,” which is frequently observed in several species and in non-human primates in particular (see Tomasello, 1999). By emphasizing the *consensual* character of this coordination I highlight two key aspects: first, that the relation between the observed interdependent behaviors would not be observed without a specific ontogenetic history, and second, that this coordination occurs as the *spontaneous* consequence of coupling. Although the term “consensual” employed by Maturana can evoke agreement and may therefore be perceived by some as ambiguous, the proposed definition should clarify its meaning in the context of a bio-logical approach. Furthermore, it should be clear that the emergence of consensual coordination is not a consequence of a deliberate, planned strategy, nor does it include goal directedness; rather, the establishment of consensual coordination allows individuals to successively draw on an already established “consensual domain” of coordination patterns, in order to operate “strategically.” Taking this definition into account, “coordination” will hereafter refer only to consensual coordination.

With that said, if we focus on interaction and consensual coordination alone, we cannot entirely explain how language and complex human sociocultural practices can emerge. This becomes clear as soon as we note that, from a bio-logical viewpoint, coordination cannot be seen as a communicative setting or “information transmission.” It would be misleading to speak of “communication” in order to account for animal coordination. This would mean that the conduct of the individuals involved “conveys a message” which refers to circumstances related to the message’s emission, “as if what determines the course of the interaction were the meaning and not the dynamics of structural coupling of the interacting organisms” (Maturana and Varela, 1992: 207). Consensual coordination does not rely on this informational model. No “information” is exchanged and no object can be denoted or *observed* by the interacting individuals. Any alleged exchange of signals between coordinating individuals is only a description of the interaction made by the observer (Maturana and Varela, 1980).

We must still wonder which specificity inherent to human coupling gives rise to language, compared to other modes of living in the biosphere where language is apparently absent. To explain the emergence of human cultural and linguistic phenomena, it is therefore necessary to make explicit the specific feature of human domain of consensual coordination.

## RECURSIVE CONSENSUAL COORDINATION: LANGUAGE AND HUMAN JOINT ACTIVITIES

Given this definition of consensual coordination between interacting individuals, I would argue that a bio-logical explanation of language and joint activity can be provided. In line with the previous considerations, this explanation must trace language’s constitutive conditions to the bio-logics of living systems. In keeping with Maturana (1988), our question could be formulated as follows: under which circumstances within the history of interactions between living beings can language emerge? Or, in other words, how can we explain linguistic activity as a class of phenomena related to structural coupling, and therefore as a consequence of a specific history of coexistence between living beings? This is an epistemological question that must first be answered from a theoretical standpoint.

Social interaction is fundamental in species for which individual ontogeny occurs as a part of a network of co-ontogenies brought about through consensual coordination. In human interactions, it is the emergence of *recursion* within the consensual domain that gives rise to the *classes* of inherently social phenomena that we distinguish as language, communication, and more generally, human sociocultural practices. *Recursive consensual coordination* is, in effect, the generative mechanism we were looking for. Building on Maturana’s work, I choose to define *languaging* as *a process based on recursive consensual coordination of individuals’ interrelated operations, taking place in the interindividual relational domain.* Minimal languaging appears in the domain of interaction as soon as individuals operate a coordination which takes place, recursively, “at the top” of their historically established domain of coordination. The new classes of operations that one can thereby distinguish still consist of consensually interrelated operations. However, they differ from those based on “flat” consensual coordination in that they only take place through a recursive process which draws on the history of other coordinated operations brought about by the individuals in prolonged, intimate coexistence.

To clarify the power of recursive coordination, it is best to see an example of how it functions. Let us consider a “flat” human coordination such as the passing of toys between an infant and his caregiver. This activity presents many aspects of a coordination framework that we can observe in other species. However, a new framework appears if the infant and his caregiver bring about a new coordination by recursively drawing on the pre-established one as an operational basis; i.e., when activity such as the play of passing toys allows the emergence of a new activity that includes the *request* to pass said objects. The circumstances are similar but we can now observe a new class of phenomena. Vocalization, gestures, movements, and the other interrelated operations are now elements of a recursive consensual coordination that is identifiable as a new activity. This new class of doing things together cannot be reduced to the previously established class; however, its possibility relies precisely on this previously established class.

This basic example shows that the process of languaging constitutes an astounding expansion of individuals’ operational relational matrix, and that it allows the generation of new classes of interrelated operations that are bio-logically possible only through recursion. Importantly, these classes of operations constitute our human doings; they coincide with our “doing things together” in coexistence as different types of joint activities. Moreover, because of the multiplying character of recursivity, new coordination can occur recursively in the flow of “doing things with others.” The flow of languaging should therefore be understood from within the mutual operational-relational interdependencies which it brings about. This flow of coordination extends beyond isolated occurrences of coordination: individuals’ respective operational spheres (including our behavioral, mental and emotional dimensions) remain interdependent beyond the event of coordination. Ontogenetically, the languaging flow sets a matrix of interdependence within which all our operations as human beings exist. “Doing things with the others” through recursive consensual coordination can therefore be considered as the invariant organization of the systemic dynamic of human structural coupling. In other words, languaging constitutes a species-specific feature of the mode of living through which we human beings exist as a distinct class of organisms. This mode of living constitutes the human “ontogenetic phenotype” (notion introduced by Maturana and Mpodozis, 2000; see, e.g., Maturana and Verden-Zöller, 2008); or to put it another way, the core feature of our “developmental system” (Oyama, 2000; Oyama et al., 2001).

Although it is not possible to develop these notions at length within the limits of this article, it is important to show the theoretical implications of an approach in terms of languaging compared to other conceptions of language. What we call “language” coincides with constitutive *elements of coordination* within languaging. Language therefore belongs to the process of languaging and can be considered as a multi-scalar system of discriminant differences which allow us to bring about different forms of activities. In such as regards the complex systems of dynamic operational configurations brought about by each event of recursive coordination, these elements can be considered as “semiotic elements” precisely in that they specify different configurations of coordination. By the same token, aspects of our operation that do not result in a difference of coordination are not “semiotic elements” in relation to a given contingent, consensual domain. Undoubtedly, we can distinguish some of the more salient classes of semiotic elements within our present cultures, and we can study them using the most thorough and sophisticated systems of analyzable regularities (lexical, grammatical and phonological). At the same time, other systems of regularities relating to the event of coordination can now be taken into account: gesture, prosody, conversational turns etc. (e.g., Kendon, 1990; McNeill, 1992; Schegloff, 2007). Nevertheless, all these systems of regularities do not explain languaging themselves, nor do they exhaustively describe the operational architecture underlying recursive coordination.

In several aspects, the explanation of languaging allows us to embrace the dialogical, actional view of language as opposed to an internalist, monological view (Linell, 2009). In keeping with the distributed approach to language (Cowley, 2007, 2011; Thibault, 2011), it should be noted that the event of coordination is a co-constructed dynamic that engages the embodied organism and occurs in real-time interactivity. Such a dynamic unfolds on extremely fast time-scales, measurable in fractions of a second. Meaning is directly inherent to the flow of recursive coordination and to its contextual operational architecture within each interactive situation.

Here I would like to emphasize that by identifying “recursive consensual coordination” as the generative mechanism underlying such a real-time, interactional process, we can understand what makes it unique in comparison to other kinds of “flat” coordination. Importantly, since it is operationally grounded on the bio-logics of structural coupling, languaging can be traced to interaction and coordination, yet it constitutes phenomena whose properties are not reducible to them. Moreover, this process takes place in a flow of operational interdependence that goes beyond the setting of any single event of coordination, and whose result is the network of human practices. Also, it is clear that language cannot be considered as being either logically primary or secondary to sociocultural activities, because language and recursive coordination are necessarily co-occurrent. Although they can be analytically distinguished, human joint activity and language arise from the same process; one is not the cause of the other.

As we have previously examined, the emergence of consensual recursive coordination does not require any previous agreement between interacting individuals. Rather, such coordination relies on the congruent transformation of our operational spheres during the process of living together, and it is a systemic, spontaneous result of this process. Recursive coordination does not therefore require agreement, or previous understanding; on the contrary, it is the condition by which agreement and understanding can arise. In fact, coordination does not even presuppose cooperation, since cooperation refers to the configuration of emotionning within which a given coordination is brought about. Even though cooperative coordination is crucial to human mode of life, what is proposed here is not an irenic vision of interaction; it includes all antagonistic forms of coordination (negotiations, conflicts) in as much as all these forms do not invalidate but rather intrinsically confirm the consensual character of coordination, along with the constitutive interdependence between individuals’ operational spheres. This occurs as *conversation*. What I refer to as “conversation” is a flow of languaging where individuals operate a recursive coordination which draws on the consensual distinction of the configuration of interrelated operations brought about by a previous occurrence of recursive coordination. For example, in conversation we can refuse or negotiate the “communicative actions” enacted (or “projected”) by others, actions that by definition specify a certain immediate or future effective interrelation between the operational sphere of others and our own. As a result, conversation allows us, by operating in languaging, to modulate or to change the course of the dynamic flow of our operational interdependence. Since this shift in the flow of languaging occurs through recursive coordination, it does not disintegrate the interrelation between our operational spheres, but allows an expansion of it while remaining within the realm of languaging. The same is true for such events as misunderstandings (or lack of understanding), that can be “repaired” through recursive coordination. Conversation provides the possibility of a fully human reciprocity, which in turn makes it possible to preserve languaging by languaging. Without conversation, our interactions would only be the accumulation of simple sequences of recursive coordination. Finally, conversation represents an immensely complex evolution compared with the phenomena brought about by “flat” coordination. I would go so far as to say that conversation is one of the fundamental aspects of our living-through-languaging.

## INTEROBJECTIVE DISTINCTIONS AND THE EMERGENCE OF OBSERVING

Having introduced recursive consensual coordination as the generative mechanism of language and joint activity, I need to make explicit another fundamental aspect of languaging. The following should further clarify the relevance of the bio-logical approach in order to overcome cognitivist accounts of the emergence of social interaction and joint activity, such as Tomasello’s. The spectatorial position that cognitivists ascribe to interacting individuals implies that they engage in the *observation* of objects, persons, intentions, “shared knowledge” and “common ground.” However, this observation cannot bio-logically precede recursive coordination and therefore cannot be a precondition of language and joint activity. To the contrary, I will show that such an operation of *observing* is generated precisely through languaging.

As claimed earlier in this paper, non-human animal interactions do not and could not take place by “referring to objects.” However, we should now explain how we as human beings refer to the circumstances related to our operation. To this end, it is necessary to define what is intended here as an object. Within the presented epistemological framework, objects are dynamic operational configurations related to recursive coordination and therefore to our relational operation. While objects are admittedly constituted through the operations of each of us as single individuals, their constitution relies on recursive coordination with others. More specifically, I consider that objects are the *sine qua non* operational condition for recursive coordination. Recursive coordination is brought about by taking a given configuration of interrelated operations as the operational basis for a further coordination. These configurations of operations remain obscured to the individuals, who only operate different kind of distinctions: “Objects arise in language as operations of coordinations of coordinations of doings that stand as coordinations of doings about which we recursively coordinate our doings as languaging beings” (Maturana, 2002: 28).

From a cognitive point of view, objects depend on operating consensual “interobjective” distinctions, that is to say, distinctions related to the configuration of interrelated operations which bring about a recursive consensual coordination. Ontogenetically, the process of languaging leads to the routinization of distinguishing objects (entities, relations, processes). This epistemological explanation implies that, for the individuals, objects are as experientially present and real as the operations that allow them to arise, independently from the domain – physical, relational, abstract, imaginary – in which they can be classed by an observer thereafter. With regard to individuals operating recursive coordination, objects exist first as immediate configurations of operation and can then be *observed* as objects through a subsequent recursive operation, as distinctions of distinctions of distinctions.

Let us explore what I mean by *observing*. If the previous considerations are clear, we can go a step further and consider what happens when individuals start distinguishing their own interobjective distinctions through recursive coordination. « Observing » becomes then possible: recursively operating on interobjective distinctions is equivalent to being mindful about the objects that are distinguished through coordination. In this regard it is important to note that observing is a process that relies on the bio-logics of living beings, to the extent that observing is a possibility inherent to the operation of the organism as a whole, provided that it can operate through recursive consensual coordination. In this light, while observing is admittedly possible only under some specific conditions (with a given phylogenetic trajectory and an ontogenetic history of coexistence while doing things together through languaging), it can be explained as a bio-logical operation without basing it on any other principle or functional device. By making us distinguish our own distinctions in terms of entities, experiences and feelings, observing is therefore another key element in the explanation of the sociocultural practices that characterize the human mode of life. In effect, it is through the operation of observing that description-making, development of narrative skills and reflection become possible. These operations draw on the process of distinction of objects arising in recursive coordination, and on its increasing recursive complexity. Furthermore, as we learn to operate distinctions through the practices within which objects exist, these objects can be operated independently of the single occurrences of interaction. This means that they are gradually embodied in the relational operation of structural coupling to our medium and are operated recurrently in the process of making sense of daily human life – even during solitary activities.

Virtually all configurations of operations can become objects in the process of languaging and therefore expand the interindividual domain of objects and practices. More generally, we are dealing with what Maturana would call an “interobjective domain” (2000, 2005), which includes both observed and non-observed objects, and is constitutively open to dynamic expansion and change, since it is strictly contingent on historical and situated circumstances of coordination. This being said, it is clear that the term “interobjective domain” relates to an abstraction that one can make of a network of dynamic languaging flows. These flows always take place in an ever-changing present during the course of interactions within a given network of human beings, and follow a not-pre-established drift which draws on an inherently peculiar, cultural history of recursive coordination. It should be remarked that the notion of “interobjective domain” can be partially assimilated into that of “common ground” (Clark, 1996; Tomasello, 2008), meaning that of common knowledge, assumptions, and norms “shared” by individuals; but only if we consider the latter from a non-intellectualist, non-spectatorial standpoint. The notion of “interobjective domain” refers to the matrix of potential configurations of coordination operable by individuals through languaging, at a given moment in their ontogenetic history.

We can now understand why languaging makes it possible for human beings to reference entities and events. Since objects are the operational condition for languaging, it follows that interactions not relying on recursive consensual coordination (such as the interactions existing between individuals of other species) also do not entail the constitution of interobjective domains. This should not be surprising, as modes of living which do not include “operating and observing objects” are clearly just as viable and adaptive for those organisms which preserve structural coupling with their medium. Where there is languaging, there are language, objects and human sociocultural activities. Where one does not exist, neither can the others. Language, objects and human joint activities arise together through languaging.

Logically, some epistemological consequences follow. First, there is no *original* “linking problem” which individuals would have to face in their supposed efforts to “connect” languaging to objects. Thus, we cannot ascribe to infants the putative task of connecting linguistic symbols to the entities existing in the world, which Tomasello would hope to facilitate with his hypothesis of intention-reading skills. Human beings do not resort to language as though it were a system of symbols denoting entities that exist beyond their recursive operation. The flow of interrelated operations in languaging allows us to constitute, conserve and multiply objects over generations. This argument challenges the representationalist function of language and its status as a system of “symbolic tools” that we “use,”^3^ although symbolic thinking does take place in languaging. We will later see the importance of this for language acquisition.

Second, any spectatorial account of language acquisition is inadequate. We have seen that Tomasello considers intention-reading as logically and ontogenetically primary. However, not only does the bio-logical conception of organism challenge both the mentalist and the folk-psychological assumptions behind this hypothesis (see §3); but also, based on the explanation of observing, infants cannot be the spectator of any “communicative intention,” mental state or of any other type of object before they operate interobjective distinctions. Since observing takes place in languaging as a condition for the establishment of complex forms of joint activity, it follows that observing can neither take place outside of nor before recursive coordination. The infant cannot observe any object before he begins to participate with others in specific kinds of doings and recursive coordination. When individuals observe, that is to say when they consensually distinguish objects related to the circumstances of coordination, they are already languaging.

Finally, and most importantly, this approach allows us to reconcile a non-representational conception of neurobiological processes (since, bio-logically, the nervous system does not work with symbols, representations or content), with the possibility of our human “contentful mindedness.” We, as human beings, operate objects as our cognitive way of living through languaging, often simultaneously observing some of these objects. However, it should be remarked that observing and consciousness constitute only one aspect of our otherwise noncontentful moment-to-moment operation within the flow of living. Interestingly, this explanation is congruent with Hutto and Myin’s (2013) Scaffolded Mind Hypothesis and Developmental Explanatory Thesis, according to which “ (…) all the mentality-constituting interactions are grounded in, shaped by, and explained by nothing more, or other, than the history of an organism’s previous interactions.”

## ONTOGENETIC IMPLICATIONS OF THE BIO-LOGICAL APPROACH

Let us now consider ontogenetic development, language acquisition and the emergence of sociocultural skills from a bio-logical standpoint. The key theoretical proposal is that children learn to speak by languaging. This means that children actually language before they are able to emit their first words. In some aspects, this turns Tomasello’s theory on its head.

First of all, I suggest that a clean separation between the prelinguistic and the linguistic stage does not allow us to fully grasp the trajectory across which the operational-relational, interindividual domain of the infant and his caregivers expands through recursive coordination. By beginning to operate in recursive coordination with them through joint activities very early on in his ontogeny, a child starts participating in the network of doings that constitute the culture within which his caregivers exist as human beings. This ontogenetic process opens up a multiplicity of further joint activities in daily coexistence.

A multitude of research has shown that coordination arises very early in infant-caregiver interactions, starting as a mutual co-orientation and emotional attunement (Stern, 1977; Trevarthen, 1979; Fogel, 1993b; Beebe and Lachmann, 2002; Greenspan and Shanker, 2004). As a relational process, early interactions establish the first domains of interrelation between the operational spheres of the child and his caregivers. The emotional and behavioral attunement thus generated becomes a consensual domain open to expansion in the course of recurrent interactions, including care practices and play. This consensual domain, although very rich, remains a domain of “flat” coordination, in some ways similar to that which we observe in other primates’ interactions.

However, it is precisely with the phenomena arising from joint attention episodes that the first events of languaging appear, bringing new possibilities to joint activity. The child can then coordinate his attentional focus with that of the caregiver, follow objects with his gaze in dyadic settings, and transform routines of manipulation into new classes of coordinated operations. By distinguishing objects related to patterns of coordination, he can start participating in new joint activities. To repeat what I have previously stated concerning the example of the passing of toys, satisfying a request pertains to a new class of interrelated actions that cannot be assimilated into the previously established configurations of coordination on which they depend.

The development of the child’s responsiveness to others’ doings, as well as of his own disposition to initiate an event of coordination, is to be understood as the spontaneous result of an ontogenetic trajectory. Across this trajectory, the variety of configurations of coordination in which he is able to participate gradually increase, while at the same time his structure changes in the course of his living. This challenges the idea of a sort of developmental discontinuity represented by Tomasello’s “nine months revolution,” the time in a child’s life at which intention-reading skills supposedly emerge. Although episodes of recursive coordination establish a new step in the history of coexistence, what we have here is a single process, and a single generative mechanism to explain its historical trajectory. In fact, sequences of pointing (Bates et al., 1979; Tomasello, 2008) belong precisely to some of the first events of recursive coordination initiated by an infant, building on the consensual domain of activities already established. On the one hand, pointing is an operational element of recursive coordination that relies on an operational basis of pre-established patterns of coordination. These patterns ensure the interrelation between operational spheres in certain circumstances. On the other hand, pointing provides the possibility of establishing a new class of coordination that includes the fact of reorienting the attention of the other. The latter results in the constitution of a new class of coordinated operations, meaning that when the child points, he is languaging, since recursive consensual coordination is brought about by all the operational elements that can possibly give rise to it, whether “verbal” or “non-verbal.” This initially sporadic participation in recursive coordination gradually allows the child to expand his range of activities through the process of operating on the consequence of recursive coordination with his close circle of relations. From this point on, the gradual distinction of new elements of coordination and objects occurs together with new events of recursive coordination. This process allows the child to acquire operational experience specific to languaging, and to make joint activity his domain of existence as a human being. The child himself then becomes a sociocultural *agent*.

Tomasello seems to have this process in mind when he speaks of non-verbal, prelinguistic communication as “natural communication” (Tomasello, 2008). However, the mentalist and spectatorial reformulation of events remains problematic in that it introduces intention-reading as an explanatory mechanism, not only lacking bio-logical grounding, but preventing us from grasping the fact that we are coping with one single process – that is to say, languaging. Moreover, the process that gives rise to language acquisition and sociocultural learning can be bio-logically explained without appealing to representationalist and spectatorial accounts. Although we as observers can contemplate a metadomain in which we associate elements of coordination and circumstances of interaction, we cannot ascribe to the child the cognitive task of matching objects in his world to “symbols” – a problem to which intention-reading would provide a solution. Not only does this solution require us to presuppose an inadequate epistemological framework, it also causes us to lose sight of the *interaction itself*. We then fail to fully understand language and joint activity as constitutively belonging to the same process. As Maturana argues, “Part of the difficulty in understanding the relation between language and existence rests on the view of language as a domain of representations and abstractions of entities that pertain to a different concrete domain. Yet language is not so, languaging occurs in the concreteness of the doings of the observer in his or her actual living in the praxis of living itself” (Maturana, 2002: 32).

## OBSERVING COMMUNICATIVE INTENTIONS

I have shown, based on Maturana’s work, that observing is the result of a history of interaction through languaging, and is a necessary operation for our mode of living in recursive coordination. This means that I do not need to posit any functional device for it, but only assume that our neurobiological processes are adequate for the relational–operational domain in which we human beings exist.

With regard to one of the most debated subjects of social cognition, it should be now clear why folk-psychology (understanding other’s beliefs and mental states) requires the operation of observing, and relies on the emergence of different objects that are operated gradually in infancy as the result of an ontogenetic history of coexistence in languaging. Different objects and different classes of recursive coordinated operations emerge gradually: self-consciousness and reflection (Maturana, 2005), meta-discursive skills (Taylor and Shanker, 2003; Taylor, 2012) and a language stance (Cowley, 2011) as well as the understanding of narrative practices (Hutto, 2008). All this allows the child to operate in an interobjective domain of beliefs and mental states. The important factor to be taken into account is therefore the process leading to the ontogenetic establishment of such a domain.

In this context, we can add a few words about intention-reading as presented by Tomasello. I have already made clear that the functional intention-reading infrastructure as presented by Tomasello is neither bio-logically grounded, nor required to account for “language acquisition.” The explanation for the ontogenetic emergence of social interaction, joint activity, language and objects has been provided by drawing on the bio-logical understanding of structural coupling and the process of recursive consensual coordination. However, another crucial point here is that while I have argued that intentions are not internal entities causing behavior, it remains true that adults constantly attribute intentions to each other in their daily life. From an epistemological standpoint, how should we actually explain this mutual attribution of communicative intentions?

Since intentions are not components of the living being’s structural domain, they should belong to the operational domain of interaction. If we draw on the explanation of objects and of the operation of observing, a rather different definition of communicative intention can be provided in place of the one presented in many mentalist approaches. I argue that communicative intentions are related to one of the previously introduced key features of languaging: conversation. I propose that we consider that what Tomasello, drawing on philosophy of language and pragmatics, calls a communicative intention is not an internal entity causing action, but instead can be explained as a class of objects constituting the *sine qua non* condition for conversation. These objects coincide with the interobjective distinction of the specific way in which individuals’ operational spheres would be interrelated by a given recursive coordination. In other words, “communicative intention” refers to the consensual distinction of the operational result to which a prefigured coordination would lead. For example, when a caregiver asks a child to fetch a toy, the communicative intention is the particular operational interrelation between the caregiver’s and the child’s operational spheres, which must be brought about in order for that specific event of coordination to be realized. However, for a communicative intention to exist it has to be operated. In the present case, the communicative intention arises as an immediate interobjective distinction when the child and his caregiver consensually operate a recursive coordination (i.e., the negotiation of the request) that modifies the prefigured trajectory of the operational interrelation (the request projected by one of them). The interobjective distinction of communicative intention is therefore the operational basis for the emergence of conversational classes of coordinated operations, such as negotiation.

Put differently, as an observer, I use the term “communicative intention” to identify a contingent interobjective distinction that is not required for a single sequence of coordination, but that rather makes possible a flow of recursive coordination (such as a conversation). These distinctions, initially operated in an immediate way by the child during his conversation with others, and only later recursively observed, can be subsequently named through a new recursion – for example, in the case of a given communicative action which individuals ascribe to each other during discourse). Finally, if communicative intentions can be “objects of observing,” could intention-observing (as defined above), rather than intention-reading (as detection of mental states), be a precursor to language, or at least to conversation? The answer is logically negative. From a logical and operational point of view, infant cannot observe any object before operating recursive coordination. No previous intention-observing is necessary in order to bring about the developmental structural transformation which allows a child to converse; on the contrary, it is only by the operational experience which each individual already has of his domain of languaging that he can begin to converse. Again, observing neither precedes nor causes recursive coordination: it does not provide individuals with the know-how for the coordination, but is rather a concomitant operational condition for several classes of activities enacted through languaging. This means that intention-observing is not a precursor to language; at the same time, we can ascribe communicative intentions to others while languaging.

## CONCLUSION

The principal aim of this paper has been to contribute to studies in the domain of social cognition and interaction by introducing some considerations on the constitutive conditions of language. From an epistemological point of view, I have focused on the domain of human interaction itself and have shown that human social interaction, language and sociocultural activities arise from the same operational-relational matrix.

What I have defined as a “bio-logical” approach challenges cognitivist accounts of social engagement and coordination. In opposition to the cognitivist hypothesis proposed by Tomasello in order to explain language acquisition and joint activity, which he considers as warranted by a Cartesian infrastructure, I have suggested that we turn our attention towards the bio-logical conditions through which the operation of observing arises. As previously stated, a generative explanation for human interactional phenomena is needed. This implies, on one hand, the identification of the domain to which we can trace the phenomena to explain (in our case, linguistic activity and sociocultural practices), and on the other hand, the proposition of a mechanism that would allow the occurrence of the phenomena to explain. Such a domain is that of structural coupling between living beings, wherein interaction plays a fundamental role. A bio-logical framework allows us to see the interactional domain itself as the appropriate domain for explaining human interactivity through the lens of “consensual coordination.” In keeping with the work of Maturana, the proposed mechanism is that of recursive consensual coordination, which can be seen as the organization underlying all linguistic activity, and more generally, human doings. By the same token, it has been possible to show the emergence of the operation of observing along with its implications in human development. Observing, self-consciousness and mindedness are human forms of existing in the operational-relational domain, and they therefore cannot be reduced to any subpersonal infrastructure.

Throughout this paper, I have also summarized the reasons for avoiding the assumption that, ontogenetically, intention-reading is a prerequisite for engaging with others in social and linguistic activities, and have provided arguments precluding such a characterization. Along with the arguments for a bio-logical understanding of language and interaction, I have developed arguments against Tomasello’s hypothesis of intention-reading as the precursor of language. On one hand, I have argued that the bio-logical understanding of organism allows us to reject both mentalist explanations and folk-psychological assumptions (see §2 and §3). On the other hand, I have shown that language is not a symbolic toolset and cannot not be considered as secondary to the establishment of joint activities, because it is a constitutive element of each event of recursive coordination (§5 and §7). Furthermore, the spectatorial stance that is implied by any sort of intention-reading skills would ultimately require the operation of observing, which can arise only through languaging and cannot therefore be its precursor (§6 and §8).

The bio-logical approach has some implications for the study of social interaction and joint activity. First, it is precisely because of our ontogenetic trajectory of structural transformation that we, as individuals developing in languaging, can operate congruently to what an observer could describe as the properties of our culturally situated system of coordination, and then, recursively and through reflection, elaborate strategies and follow individual or joint goals congruent to our coordination experience. Second, in order to explain coordination we cannot trace it to such notions as communication, cooperation, symbols or intentions which we use to refer to aspects of the process of coordination itself, and cannot therefore give rise to it. Rather, it is necessary to reveal the bio-logical framework within which the phenomena related to the same notions take place. This is one of the reasons why we cannot rely on a functionalist conception of language as a tool used for extra-linguistic transactions, as activities that could occur without or before languaging; this manner of proceeding confuses the way we make sense of our doings in languaging with the genesis of languaging. Third, it is not so much that language has an important impact on human agency and cultural life, but rather, languaging *is* human agency. As said before, the operations that give rise to recursive coordination are the constitutive, discriminant elements that configure a given event of coordination as such. We do not “use” these elements; rather, we enact them throughout the operational flow of coordination, although in some cases, by observing and therefore by constituting them as objects, we can consider that we are using them to produce a certain effect.

Finally, by recognizing recursive consensual coordination as an invariant organization of human interactional dynamics, it becomes possible to understand different classes of phenomena, from language acquisition to all kind of sociocultural practices, as resulting from a single process. These phenomena remain to be studied in detail within their own domains, but the bio-logical explanation of languaging steers us towards a wider scope of understanding social interaction, and our specific mode of “doing things with others”.

## Conflict of Interest Statement

The author declares that the research was conducted in the absence of any commercial or financial relationships that could be construed as a potential conflict of interest.

## Footnotes

1 According to Tomasello, “shared intentionality,” presented as the mutual acknowledgment of joint commitment and joint intentions between interacting individuals, is a necessary condition for the realization of human practices, since they all supposedly involve “sharing of psychological states” in a cooperative goal.

2 In this paper I will maintain a distinction between the terms “interaction” and “structural coupling”; while employing the latter to refer to the bidirectional, constant mutual triggering between organism and its biotic and abiotic medium, I reserve the use of the former to refer to delimited events where a given sequence of interlocked operations is distinguishable between two or more organisms.

3 As Maturana argues: “It is because we human beings find ourselves operating in language as our natural manner of being that we live *language as if this were a transparent instrument by means of which we coordinate our behaviors in the distinction and handling of objects* – as if these existed independently from what we do with them – and we do not see what we are doing as we language. Because we live without seeing what we do as we language, we do not see that what constitutes our languaging is our living in a recursive flow in coordinations of coordinations of doings, and that objects arise as tokens of coordinations of doings that obscure the doings they coordinate in this recursive flow.” (Maturana, 2000: 462; italics are mine).
